# Reaction Rate Rules of Intramolecular H-Migration Reaction Class for R^I^OR^II^OO·Radicals in Ether Combustion

**DOI:** 10.3390/molecules29184387

**Published:** 2024-09-15

**Authors:** Xiaohui Sun, Zerong Li

**Affiliations:** 1 School of Energy Engineering, Shanxi College of Technology, Shuozhou 036000, China; 2 College of Chemistry, Sichuan University, Chengdu 610064, China

**Keywords:** intramolecular H-migration reaction, reaction rate rules, isodesmic reaction method (IRM), reaction class transition state theory (RC-TST)

## Abstract

The intramolecular H-migration reaction of R^I^OR^II^OO· radicals constitute a key class of reactions in the low-temperature combustion mechanism of ethers. Despite this, there is a dearth of direct computations regarding the potential energy surface and rate constants specific to ethers, especially when considering large molecular systems and intricate branched-chain structures. Furthermore, combustion kinetic models for large molecular ethers generally utilize rate constants derived from those of structurally similar alcohols or alkane fuels. Consequently, chemical kinetic studies involve the calculation of energy barriers and rate rules for the intramolecular H-migration reaction class of R^I^OR^II^OO· radicals, which are systematically conducted using the isodesmic reaction method (IRM). The geometries of the species participating in these reactions are optimized, and frequency calculations are executed using the M06–X method in tandem with the 6–31+G(d,p) basis set by the Gaussian 16 program. Moreover, the M06–2X/6–31+G(d,p) method acts as the low-level ab initio method, while the CBS–QB3 method is utilized as the high-level ab initio method for calculating single-point energies. Rate constants at the high-pressure-limit are computed based on the reaction class transition state theory (RC-TST) by ChemRate program, incorporating asymmetric Eckart tunneling corrections for intramolecular H-migration reactions across a temperature range of 500 to 2000 K. It was found that the isodesmic reaction method gives accurate energy barriers and rate constants, and the rate constants of the H-migration reaction for R^I^OR^II^OO· radicals diverge from those of comparable reactions in alkanes and alcohol fuels. There are significant disparities in energy barriers and rate constants across the entire reaction classes of the H-migration reaction for R^I^OR^II^OO· radicals, necessitating the subdivision of the H-migration reaction into subclasses. Rate rules are established by averaging the rate constants of representative reactions for each subclass, which is pivotal for the advancement of accurate low-temperature combustion reaction mechanisms for ethers.

## 1. Introduction

The burgeoning demand for energy, coupled with the limited supply of fossil fuels and their detrimental environmental consequences, has directed scientific inquiry towards the pursuit of alternative fuels as a means to gradually supplant conventional fossil-based energy sources. Biofuels, as an emerging category of alternative fuels, have increasingly captured the spotlight due to their renewable characteristics and their capacity to mitigate net CO_2_ emissions [1,2,3,4]. A thorough comprehension of the combustion dynamics of biofuels is essential for the optimal utilization of these advanced renewable energy sources. Typically, investigating the combustion process of biofuels (and their blends) through experimental methods is costly and time-intensive. However, theoretical research provides a cost-effective and efficient means to simulate the combustion process of fuels across a broad spectrum of pressures, temperatures, and equivalence ratios, based on a detailed combustion reaction mechanism [2]. The accuracy of these simulations hinges on the completeness of the reaction pathways and the precision of the thermodynamic parameters within the combustion reaction mechanism. Nevertheless, a comprehensive reaction mechanism encompasses hundreds of species and thousands of elementary reactions, which are typically constructed using automated mechanism generation, tailored to various reaction classes [5]. Additionally, the reaction mechanism within mechanism programs is commonly segregated into two components: the C0–C4 core mechanism and the C5 and above extension mechanism (which includes reactions involving larger species), both generated in accordance with reaction rate rules [6,7,8,9]. When there exists a substantial disparity in energy barriers within a reaction class, it is further subdivided into distinct reaction subclasses, with specific rate rules assigned to each subclass [10].

**Scheme 1:**
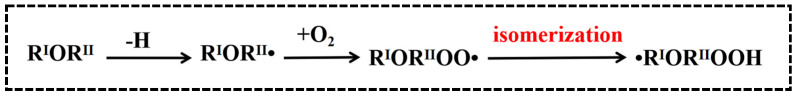
Reaction path for ethers at low temperature [3].

Numerous studies have been conducted on the reaction mechanisms of hydrocarbon fuels to date [11,12,13,14]. In comparison, research into the combustion chemistry of biofuels is relatively sparse compared to that of hydrocarbon fuels. Ethers, a type of biofuel, have a general formula of R^I^OR^II^, wherein R^I^ and R^II^ represent alkyl groups that may be of identical or distinct structures. The low-temperature oxidation process of ether fuels, as shown in Scheme 1, is similar to the low-temperature reaction mechanism of alkanes. To differentiate the radicals generated from the oxidation of alkanes, the expression method for radicals produced during the oxidation of ethers in this study employs the same formula as that used by Tommaso et al. [3]. Here, alkyl radicals (R·), alkyl-peroxy radicals (ROO·), and hydroperoxyl-alkyl radicals (QOOH) are denoted as R^I^OR^II^·, R^I^OR^II^OO·, and ·R^I^OR^II^OOH, respectively. The detailed one-step oxygenation reaction process is illustrated in Scheme 1. Initially, ethers are consumed via abstraction reactions by hydrogen atoms or hydroxyl radicals, resulting in the generation of R^I^OR^II^· radicals. The activation energy for H-atom abstraction is low, and the low-temperature oxidation activity is higher than that of alkane fuels with the same chain length, owing to the presence of the oxygen atom in R^I^OR^II^· radicals, which weakens the α-C-H bond (near the oxygen atom). Analogous to alkyl radicals, R^I^OR^II^· radicals react with oxygen molecules to form R^I^OR^II^OO· radicals, which can undergo isomerization reactions leading to the formation of ·R^I^OR^II^OOH radicals. This class of reactions is not only crucial in the oxidation of ethers but also in the analogous process for hydrocarbons, and it is also one of the factors contributing to the negative temperature coefficient (NTC) behavior of ethers in low-temperature combustion.

The oxidation mechanism of ether fuels plays a key role under atmospheric conditions, and it has also been reported in some studies in the literature [15,16,17,18]. For instance, Burke et al. [17] conducted measurements on the ignition delay and sensitivity analysis of dimethyl ether at high pressures. They discovered that intramolecular H-migration reactions involving R^I^OR^II^OO· radicals are significant in low-temperature combustion chemistry. Nevertheless, high-pressure-limit rate constants for this class of reactions are scarce, with only a few available for small molecular systems of R^I^OR^II^OO· radicals. Dimethyl ether (DME) and diethyl ether (DEE) are the most extensively studied members of this class, both experimentally and theoretically. Adamo et al. [18] proposed an autoxidation reaction mechanism for diethyl ether (DEE) using density functional theory (DFT) and the B3LYP/6–31+G(d,p) method to calculate the rate constants for reactions. However, there is a lack of direct calculation of the potential energy surface and rate constants for ether fuels, particularly for large molecular systems and complex branched-chain ethers, which possess a higher calorific value and are more suitable as direct fuels [19]. Due to the limited availability of accurate ab initio methods for ethers, the rate constants in combustion kinetic models of ethers typically employ rate constants derived from alcohols or alkane fuels with similar structures. Several theoretical studies have investigated the role that the different groups play in the H-migration reaction of peroxy radicals, such as Vereecken et al. [20] and Otkjær et al. [21]. The results show that when the carbon atoms connected by the extracted hydrogen atoms are replaced by different substitutes, the rate constants are very different, so it is necessary to calculate the rate constant accurately for ether fuels.

It is widely acknowledged that high-level quantum chemistry methods, including the Gaussian-n method [22,23,24,25], High accuracy Extrapolated Ab initio Thermochemistry (HEAT) method [26], Complete Basis Set (CBS) method [27,28], and the Coupled Cluster theory with Single and Double excitations and a quasi-perturbative treatment of connected Triple excitations [CCSD(T)] method, are extensively employed for calculating electronic energies with chemical accuracy of roughly 1 kcal/mol [29]. Nonetheless, these high-level ab initio methods are best suited for small- to medium-sized molecules and are computationally intensive. Consequently, accurately determining single-point energies for large molecular systems remains a formidable challenge. Building upon our prior research [30], we have applied the isodesmic reaction method (IRM) in conjunction with the reaction class transition state theory (RC-TST) to compute rate constants for concerted elimination reaction classes of hydroperoxyl-alkyl-peroxyl radicals, achieving results closely matching those from high-level methods. In the present study, we utilize the isodesmic reaction method in combination with reaction class transition state theory to determine accurate energy barriers and rate constants at high-pressure-limits for intramolecular H–migration reaction of R^I^OR^II^OO· radicals. Within reaction class transition state theory, a representative reaction of smaller size within the reaction class is chosen as the reference reaction, while other reactions within the class are considered as target reactions. Then, the high-pressure-limit rate constants for the target reactions are computed using a low-level ab initio method, and subsequently, these results are refined through the isodesmic reaction correction scheme.

The objectives of this study are as follows: (1) To supply high-precision reaction barriers and rate constants at high-pressure-limits for the intramolecular H–migration reaction of R^I^OR^II^OO· radicals. (2) To furnish reaction rate rules at high-pressure-limits for the intramolecular H–migration reaction of R^I^OR^II^OO· radicals. These findings will lay a theoretical groundwork for the development of more reliable combustion mechanisms for ether fuels at low temperatures. (3) To systematically investigate and compare the rate constants for the intramolecular H–migration reaction of ROO·, R^I^OR^II^OO·, and HOROO· radicals, thereby offering theoretical insights to enhance the application of biofuels.

## 2. Results and Discussion

In this study, we have selected a total of 41 reactions involving R^I^OR^II^OO· radicals of C2–C8, as detailed in Table 1. These reactions are categorized into four classes based on the distance between the carbon atom that the hydrogen atom is shifted from, which determines the ring size of transition states. To reduce the uncertainty of the rate rules for these reaction classes, they are further subdivided into three subclasses, based on the type of carbon atoms, which connected the migrating hydrogen atoms. Specifically, the carbon atom that is connected with three hydrogen atoms is defined as the “p” site, whereas a carbon atom that is connected with two hydrogen atoms and one hydrogen atom is defined as the “s” and “t” site, respectively. Consequently, the 41 intramolecular H-migration reactions are classified into the following subclasses: 1,n-H(p), 1,n-H(s), and 1,n-H(t), where n equals 3, 5, 6, or 7. In the text, tables, and figures, a radical site is denoted by a bullet symbol.

**Scheme 2:**
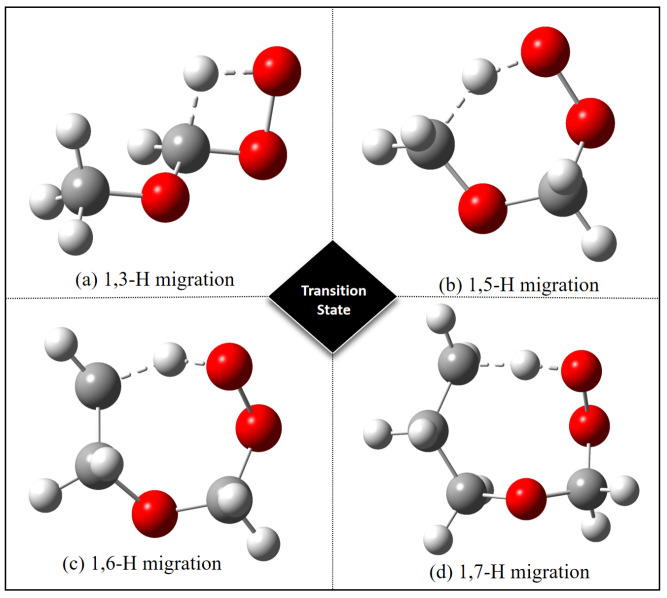
The geometry structure of transition states (TSs) for different reaction classes. (**a**)1,3–H migration (**b**) 1,5–H migration (**c**)1,6-H migration (**d**)1,7–H migration.

The reaction centers for the transition states of various reaction classes are depicted in Scheme 2. It is evident that the reaction center for these transition states forms a ring structure, and the 1,n-H migration reactions proceed through transition states with (n + 1) member-ring sizes.

### 2.1. Energy Barriers

#### 2.1.1. Validation of the Energy Barriers by the CBS–QB3 Method

**Figure 1:**
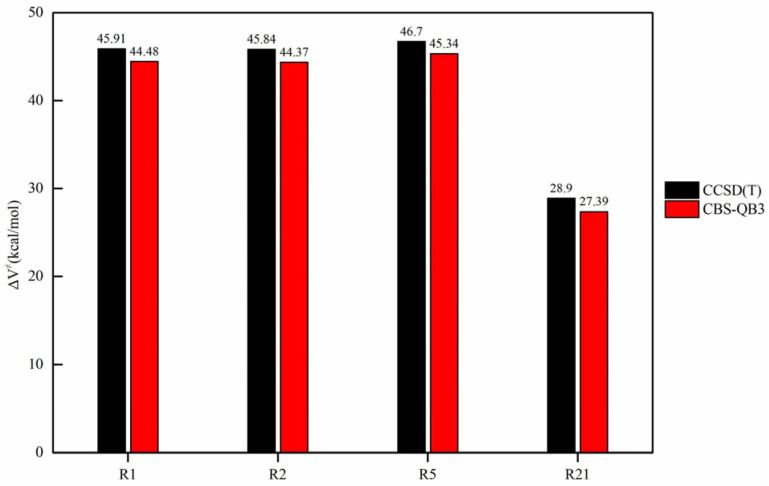
Comparison of the energy barriers between the CCSD(T)/cc-PVTZ and CBS–QB3 method.

The precision of energy barriers is crucial for ensuring the reliability of rate constants. High-precision computational methods, such as the CCSD(T) method, are capable of calculating these energy barriers and reaction enthalpies with an accuracy of 1 to 2.0 kcal/mol [29]. However, this method is time-consuming when calculating electronic energies and is not practical for large molecular systems. Consequently, the CBS–QB3 method has been employed as the high-level ab initio method in this study. To confirm the accuracy of the energy barriers computed by the CBS–QB3 method, reactions R1, R2, R5, and R21 from Table 1 are randomly selected for comparison with the benchmark CCSD(T)/cc-PVTZ method. The data presented in Figure 1 illustrate that the energy barrier discrepancies between the CCSD(T)/cc-PVTZ and CBS–QB3 methods for reactions R1, R2, R5, and R21 are 1.43 kcal/mol, 1.47 kcal/mol, 1.36 kcal/mol, and 1.51 kcal/mol, respectively. These values fall within the range of chemical accuracy, which is typically defined as 1 to 2 kcal/mol [29,31]. These findings suggest that the energy barriers obtained using the CBS–QB3 method are acceptable. Furthermore, the energy barrier difference of 1.51 kcal/mol results in a reaction rate constant ratio of 3.12 at the standard ignition temperature of 500 K, which increases by a factor of 3.68 at the standard combustion temperature of 1500 K. Both values fall within the order of magnitude and are within the margin of error.

#### 2.1.2. Energy Barriers for R^I^OR^II^OO· Radicals

In this study, the first reaction within each subclass has been chosen as the reference reaction, with the remaining reactions in each subclass considered as target reactions. The disparities in energy barriers and reaction enthalpies between the low-level M06–2X method and the high-level CBS–QB3 method for the reference reactions of each subclass are detailed in Table S1 of the Supporting Information. The energy barriers for all reactions presented in Table 1 have been computed using the M06–2X method, incorporating isodesmic reaction corrections, and are listed in Table S2 of the Supporting Information. Utilizing the data from Table S2, the average value and maximum deviation of energy barriers for each subclass are compiled in Table 2. Upon Table 2, with the exception of the 1,3-H migration reaction class, the maximum deviations in energy barriers for 1,5-H, 1,6-H, and 1,7-H are 7.19 kcal/mol, 6.85 kcal/mol, and 6.70 kcal/mol, respectively. When the reaction class is further subdivided into reaction subclasses, the range of maximum deviations in energy barriers spans from 0.23 to 2.69 kcal/mol. Consequently, it is both logical and essential to categorize the reaction class into distinct reaction subclasses.

To verify the precision of the correction scheme for energy barriers, a selection of 11 representative target reactions from Table 1 is made by contrasting the energy barriers computed using the isodesmic reaction method against those calculated by the CBS–QB3 method. The results are detailed in Table 3. Inspection of Table 3 reveals that the discrepancies in energy barriers between the isodesmic reaction method and the CBS–QB3 method range from −0.70 to 0.56 kcal/mol, figures that fall within the bounds of chemical accuracy [29]. This suggests that the energy barriers determined by the isodesmic reaction method are indeed acceptable.

### 2.2. Reaction Enthalpies

In this study, the enthalpies associated with intramolecular H–migration reaction for R^I^OR^II^OO· radicals are calculated employing the isodesmic reaction method. To ensure the reliability of the correction scheme embedded within the isodesmic reaction method, 10 reactions are randomly selected from Table 1 for verification. Both the CBS–QB3 method and the isodesmic reaction method are utilized to compute the reaction enthalpies for these selected reactions. The results are detailed in Table 4. As can be observed from Table 4, the deviations for reaction enthalpies calculated by the isodesmic reaction method in conjunction with the CBS–QB3 method range from −0.18 to 0.32 kcal/mol, a range that falls within the bounds of chemical accuracy (1~2 kcal/mol) [29]. Furthermore, for reaction R10, the reaction enthalpy computed by the isodesmic reaction method in this study amounts to 9.77 kcal/mol, which is in close proximity to the 9.30 kcal/mol reported by Curran et al. [32]. Therefore, it is concluded that the correction scheme of the isodesmic reaction method is viable for calculating precise energy barriers and reaction enthalpies for large molecular systems, achieved by adjusting values from low-level ab initio methods. The reaction enthalpies for all reactions are listed in Table S2 of the Supporting Information.

### 2.3. Rate Constants and Rate Rules at High-Pressure-Limit

In this study, the high-pressure-limit rate constants are performed with the ChemRate program [33]. Subsequently, these rate constants are fitted to the modified Arrhenius equation [11]: $k=AT^{n}\exp(-E/RT)$ in the form of (*A*, *n*, *E*) over a temperature range of 500 to 2000 K, in increments of 100 K. Here, *A* represents the pre-exponential factor, *E* denotes the activation energy, and *R* is the gas constant.

The rate rules for each subclass are derived using expression (1) and are presented in the format (*A*, *n*, *E*), as listed in Table S3 of the Supporting Information.

(1) $$k_{rule}^{T}=\frac{1}{N}\sum_{i=1}^{i=N}k_{i}^{T}$$

Here, *k_rule_* and *k_i_* represent the average rate constant and the rate constant of any reaction *i* within a subclass at temperature *T*, respectively. The total number of reactions within the reaction subclass is denoted by *N*.

#### 2.3.1. Comparison of Rate Constants by Isodesmic Reaction Method with CBS–QB3 Method

**Figure 2:**
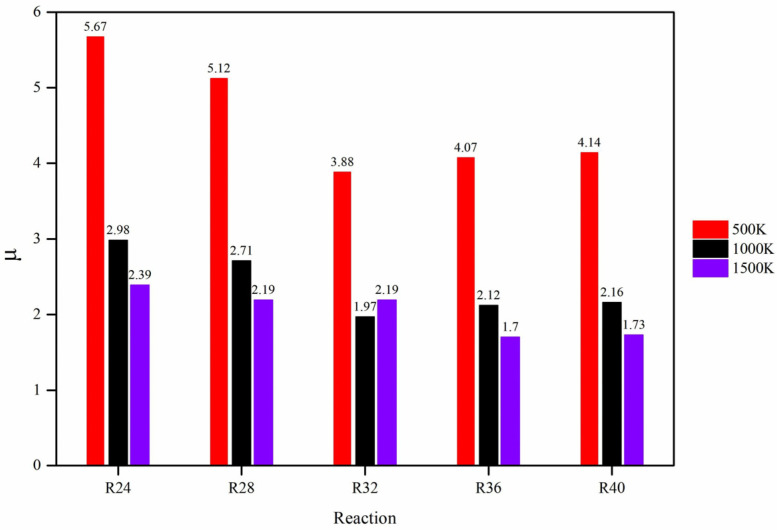
Comparison of the rate constants between the CBS–QB3 method and isodesmic reaction method at 500 K, 1000 K, and 1500 K.

In order to evaluate the reliability of the rate constants derived from the modified scheme using the isodesmic reaction method, the rate constants computed by both the isodesmic reaction method and the CBS–QB3 method for reactions R24, R28, R32, R36, and R40 from Table 1 are compared. A ratio factor *μ* (*μ* = *k_IRM_*/*k_CBS_*) is employed to assess the deviation of rate constants across the temperature range of 500 to 2000 K. To provide a clearer insight, Figure 2 presents a comparative analysis of rate constants derived using the isodesmic reaction method and the CBS–QB3 method at temperatures of 500 K, 1000 K, and 1500 K. The data reveal that the ratios of the rate constants are consistently within the order of magnitude, suggesting that the rate constants derived from the isodesmic reaction method closely align with those yielded by the CBS–QB3 method. Consequently, the correction scheme at the M06–2X/6–31+G(d,p) level of theory can be reliably employed for calculating accurate rate constants for the reactions of interest.

#### 2.3.2. Comparison of the Rate Constants with Values in the Literature for Alkanes

In the preceding introduction, we noted that the current reaction mechanism for ether fuels frequently utilizes the rate constants of analogous alkane reactions that are devoid of specific rate constants. This approach can lead to considerable inaccuracies in the reaction mechanism of ether fuels. Consequently, we have chosen four reactions from Table 1 to assess the discrepancies in the rate constants for H–migration reactions of R^I^OR^II^OO· radicals in ethers and comparable reactions involving ROO· radicals in alkanes, as detailed by Villano et al. [7] and Bugler et al. [34]. The results are presented in Table 5. Inspection of Table 5 reveals that the rate constants at the high-pressure-limit, as calculated in our study, deviate from those reported by Villano et al. [7] and Bugler et al. [34], with the ratio varying from 1.06 to 2.11 × 10^4^ at 500 K. Therefore, the impact of the R^I^OR^II^ group cannot be ignored. It is imperative to establish rate rules at the high-pressure-limit for R^I^OR^II^OO· radicals, which are also vital for the advancement of reaction mechanisms in automated reaction mechanism generators.

#### 2.3.3. Comparison of the Rate Constants in Analogous Reaction Subclass for R^I^OR^II^OO· in Ethers with HOROO· in Alcohols and ROO· in Alkanes

**Figure 3:**
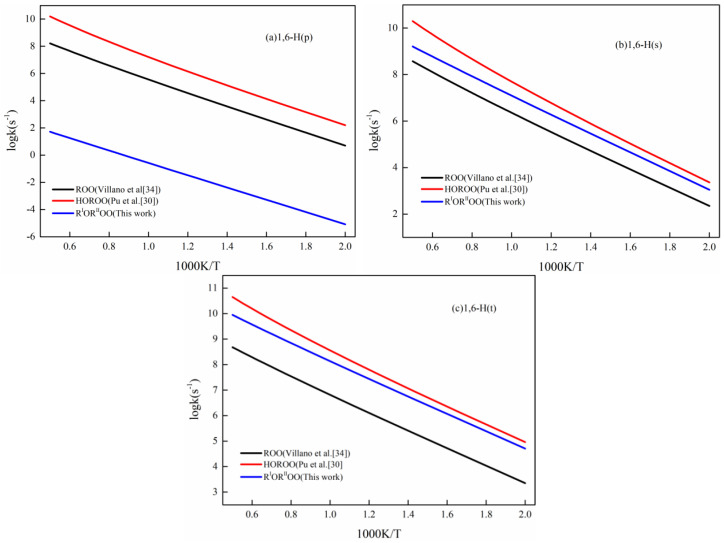
Comparison of average rate constants for 1,6 H–migration reaction of different peroxy radicals [30,34]. (**a**) 1,6-H(p) (**b**) 1,6-H(s) (**c**) 1,6-H(t).

**Figure 4:**
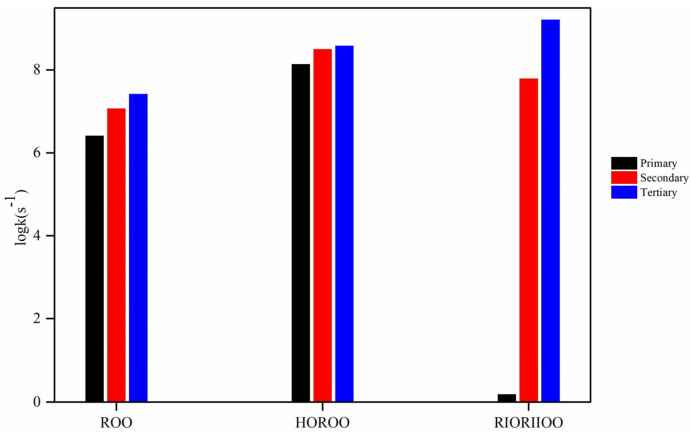
Rate constants of 1,6 H–migration reaction (1200 K) as a function of the type of carbon atoms of different peroxy radicals.

As outlined in the introduction, the prevalent reaction mechanism for ether fuels typically employs kinetic parameters derived from analogous reactions involving alcohols or alkanes. Kerschgens et al. [35] observed that the ignition properties of ether fuels diverge from those of alcohol fuels and alkanes. Furthermore, the comparison of different substitutes on the carbon atom that the hydrogen atom abstracted is conducted by Otkjær et al. [21]. Consequently, a comparison has been made between the kinetic parameters for 1,6-H migration of R^I^OR^II^OO· radicals in ethers, the HOROO· radicals in alcohols [36], and the ROO· radicals in alkanes [34], as depicted in Figure 3. Figure 3 illustrates that kinetic parameters for the same reaction subclass vary when catalyzed by different peroxy radicals. Notably, the kinetic parameters for HOROO· radicals in alcohols are the highest among the three types of peroxy radicals. In the case of the 1,6-H(p) reaction subclass, the rate constant of the hydrogen migration reaction of R^I^OR^II^OO· radicals are the smallest. For the 1,6-H(s) and 1,6-H(t) subclasses, the reactivity of the HOROO· and R^I^OR^II^OO· radicals surpass that of the ROO· radical, suggesting that the presence of oxygen-bearing groups reduces the bond energy of the C-H bond. This conclusion is consistent with the findings of Otkjær et al. [21]. Furthermore, we also contrasted the rate constants for the hydrogen migration reaction within the same peroxy group, considering the varying types of carbon atoms, as depicted in Figure 4. From Figure 4, the rate constant for the 1,6-H migration reaction of peroxy radicals with varying types of carbon atoms exhibits the following trend: tertiary > secondary > primary. This outcome aligns with the observations made by Otkjær et al. [21]. The reactions forming secondary radicals are about seven orders of magnitude faster than those forming primary radicals, and reactions leading to tertiary radicals are around 27 times faster than those leading to secondary radicals at 1200 K.

In summary, when considering the discussion on the variance of energy barriers within reaction classes as detailed in Section 2.1.2, further stratification of these classes into distinct reaction subclasses can diminish the uncertainty of the rate rules derived from the rate rule method. Concurrently, in the context of constructing the reaction mechanisms for large molecular ether fuels, it is inappropriate to employ rate constants from analogous reactions involving alkanes and alcohol fuels. Consequently, this paper proposes to formulate specific reaction rate rules for each subclass using the rate rule method, as elaborated upon in Section 2.3.4 below.

#### 2.3.4. The Rate Rules at High-Pressure-Limit for R^I^OR^II^OO· Radicals

Rate rules are crucial for constructing reaction mechanisms in automated mechanism generators. Typically, identical rate constants are assigned to reactions within the same class. However, this approach can occasionally yield significant errors, necessitating a further subdivision of reaction classes into distinct subclasses based on the varying energy barriers within each class. The high-pressure-limit rate rules for these different subclasses are detailed in Table 6. Furthermore, a ratio factor *f*, which compares the maximum rate constants to the minimum rate constants, is employed to assess the uncertainty of the rate rules within a subclass. According to Table 6, the uncertainty for the 1,n-H(p) subclass is substantial, with *f* ranging from 2.46 × 10^3^ to 4.71 × 10^3^ at 500 K and from 1.59 × 10^3^ to 2.63 × 10^3^ at 1000 K. Additionally, the uncertainty for the rate rules of the 1,n-H(s) and 1,n-H(t) subclasses falls between 1.27 to 25.2 and 1.98 to 14.20, respectively. This indicates that the rate constant derived using the reaction rate rule method exhibits a certain degree of deviation. It is essential to calculate the rate constant for each subclass precisely while considering the effect of the conformation for reactants and the tunneling effect.

## 3. Methods

### 3.1. Electronic Structure Calculation

All quantum chemical computations are performed using the Gaussian 16 program [37]. The geometries of the species involved in the reactions are optimized using the M06–2X method in conjunction with the 6–31+G(d,p) basis set. Vibrational frequencies are computed using the same method, with a scaling factor of 0.979 applied. Intrinsic Reaction Coordinate (IRC) analysis [38] is employed to ensure that the transition states are correctly connecting the minima associated with the products and reactants. The CBS–QB3 method is chosen as the high-level ab initio method for calculating the single-point energies of reference reactions in the isodesmic reaction method. This method demonstrates an accuracy exceeding 1 kcal/mol in predicting the heats of formation for a large set of molecules during the tests [39,40].

### 3.2. Calculation of the Rate Constant

Regarding the calculation of rate constants for large molecular systems, the reaction class transition state theory, developed by Truong [41,42] and their colleagues, is designed for elementary reactions within a class using a relatively low-level ab initio method. The fundamental principle of RC-TST is that both the reference reaction and any target reaction within the same class share the same reaction center. The rate coefficient *k_r_* for the reference reaction is known, having been determined experimentally or through high-level ab initio methods, whereas the rate constants *k_t_* for the target reactions are unknown and require calculation. In our previous work [30], we provided a detailed explanation of the reaction class transition state theory and defined it as the reaction class isodesmic reaction method. Here, we offer a brief description as follows:

The reference reaction and target reaction within the reaction class are established as

(2) $$A_{r}+B_{r}={\mathrm{TS}}_{r}\cdot \Delta V_{r}^{\neq }$$

(3) $$A_{t}{+B}_{t}{=\mathrm{TS}}_{t}\cdot \Delta V_{t}^{\neq }$$

where $\Delta V_{r}^{\neq }$ and $\Delta V_{t}^{\neq }$ represent the energy barriers for the reference reaction (2) and the target reaction (3), respectively. When subtracting reaction (2) from reaction (3), the resulting reaction (4) can also be regarded as an isodesmic reaction, where ΔΔ*V*^≠^ is the energy barrier for reaction (4).

(4) $$A_{t}{+B}_{t}{+\mathrm{TS}}_{r}{=A}_{r}+B_{r}+{\mathrm{TS}}_{t}\cdot \Delta \Delta V^{\neq }={\Delta V}_{t}^{\neq }-{\Delta V}_{r}^{\neq }$$

The isodesmic reaction method in the reaction class allows for the calculation of accurate energy barriers for target reactions R_t_ using a straightforward correction scheme based on reference reactions R_r_. Within this scheme, reaction (4) is treated as an isodesmic reaction, from which the following expression can be derived:

(5) $$\Delta \Delta V^{\neq }={\Delta V}_{t}^{\neq ’}-{\Delta V}_{r}^{\neq ’}={\Delta V}_{t}^{\neq }-{\Delta V}_{r}^{\neq }$$

where $\Delta V_{t}^{\neq ’}$ and $\Delta V_{r}^{\neq ’}$ represent the accurate energy barriers obtained from high-level ab initio methods or experimental data, and $\Delta V_{t}^{\neq }$ and $\Delta V_{r}^{\neq }$ denote the energy barriers calculated using low-level ab initio methods. Consequently, the following expressions can be derived:

(6) $$\Delta V^{\neq }={\Delta V}_{r}^{\neq ’}-{\Delta V}_{r}^{\neq }$$

(7) $${\Delta V}_{t}^{\neq ’}={\Delta V}_{t}^{\neq }+\Delta \Delta V^{\neq }$$

where ΔΔ*V*^≠^ represents the energy barrier value for reference reactions between the high-level and low-level ab initio methods. This value is utilized to adjust the energy barriers computed using the low-level method within the same reaction class for any target reactions, as per Equation (7). Following the correction with Equation (7), high-precision energy barriers can be derived from a low-level ab initio method, such as the DFT method. Likewise, accurate reaction enthalpies for the target reactions can be acquired using Equation (8).

(8) $${\Delta H}_{t}^{\neq ’}={\Delta H}_{t}^{\neq }+\Delta \Delta H^{\neq }$$

Here, $\Delta H_{t}^{’}$ represents the high-precision reaction enthalpies for the target reaction, and $\Delta H_{t}$ are low-level reaction enthalpies for the target reactions. Furthermore, by adjusting the approximate rate constant *k* at a low-level ab initio method in accordance with expression (9), the exact rate constant *k’* for any target reaction within the same reaction subclass can be derived.

(9) $$k^{’}=kexp\left(\frac{-\Delta \Delta V^{\neq }}{RT}\right)$$

Here, ΔΔ*V*^≠^ represents the correction scheme for energy barriers, as derived from the reference reaction in expression (6).

## 4. Conclusions

In this study, we employed the isodesmic reaction method, grounded in reaction class transition state theory, to compute precise energy barriers and rate constants for H–migration reaction involving R^I^OR^II^OO· radicals. We then constructed high-pressure-limit rate rules by averaging the rate constants of representative reactions within a subclass. Our findings lead to the following conclusions:

(1) After adjusting the energy barriers and reaction enthalpies for the target reaction at the M06–2X level using the isodesmic reaction method, the calculated values align closely with CBS–QB3 results and data in the literature.
(2) The overall reaction class exhibits a maximum deviation in energy barriers and rate constants that exceeds chemical accuracy, necessitating the subdivision of H–migration reaction into distinct subclasses.
(3) The high-pressure-limit rate constants derived in this work diverge from values in the literature for alkanes, with the ratio ranging from 1.06 to 2.11 × 10^4^ at 500 K. The presence of C-O-C bonds results in a weakening of the C-H bond adjacent to the ether group.
(4) By comparing the rate constants for ROO·, R^I^OR^II^OO·, and HOROO· radicals, we determined that the rate constants for the H–migration reaction of HOROO· radicals are greater than those for ROO· and R^I^OR^II^OO· radicals. The maximum deviation in rate constants between R^I^OR^II^OO· and HOROO· radicals span two orders of magnitude. Consequently, it is imperative to establish separate rate rules for the H–migration reaction of R^I^OR^II^OO· radicals, rather than adopting kinetic parameters from alcohols or alkane fuels with analogous reactions. Additionally, the rate constant derived using the reaction rate rule method exhibits a certain degree of deviation. It is essential to calculate the rate constant for the elementary reaction of ether fuels precisely.

## Figures and Tables

**Table 1:** Reaction equation lists.

Reaction Class	Reaction Equation
1,3-H(s)	
R1	CH_3_OCH_2_OO·→ CH_3_OC·HOOH
R2	CH_3_CH_2_OCH_2_OO· → CH_3_CH_2_OC·HOOH
R3	CH_3_(CH_2_)_2_OCH_2_OO· → CH_3_(CH_2_)_2_OC·HOOH
R4	CH_3_(CH_2_)_3_OCH_2_OO· → CH3(CH_2_)_3_OC·HOOH
1,3-H(t)	
R5	CH_3_OCH(CH_3_)OO· → CH_3_OC·(CH_3_)OOH
R6	CH_3_OCH(C_2_H_5_)OO· → CH_3_OC·(C_2_H_5_)OOH
R7	CH_3_CH_2_OCH(CH_3_)OO· → CH_3_CH_2_OC·(CH_3_)OOH
R8	CH_3_(CH_2_)_2_OCH(CH_3_)OO· → CH_3_(CH_2_)_2_OC·(CH_3_)OOH
R9	CH_3_(CH_2_)_3_OCH(CH_3_)OO· → CH3(CH_2_)_3_OC·(CH_3_)OOH
1,5-H(p)	
R10	CH_3_OCH_2_OO· → ·CH_2_OCH_2_OOH
R11	CH_3_OCH(CH_3_)OO· → ·CH_2_OCHCH(CH_3_)OOH
R12	CH_3_OCH(C_2_H_5_)OO· → ·CH_2_OCH(C_2_H_5_)OOH
1,5-H(s)	
R13	CH_3_CH_2_OCH_2_OO· → CH_3_C·HOCH_2_OOH
R14	CH_3_(CH_2_)_2_OCH_2_OO·→ CH_3_CH_2_C·HOCH_2_OOH
R15	CH_3_CH_2_OCH(CH_3_)OO· → CH_3_C·HOCH(CH_3_)OOH
R16	CH_3_CH_2_OCH(C_2_H_5_)OO· → CH_3_C·HOCH(C_2_H_5_)OOH
R17	CH_3_CH(CH_3_)CH_2_OCH(CH_3_)OO· → CH_3_CH(CH_3_)C·HOCH(CH_3_)OOH
1,5-H(t)	
R18	CH_3_CH(CH_3_)OCH_2_OO·→ CH_3_C·(CH_3_)OCH_2_OOH
R19	CH_3_CH(CH_3_)OCH(CH_3_)OO·→ CH_3_C·(CH_3_)OCH(CH_3_)OOH
1,6-H(p)	
R20	CH_3_CH_2_OCH_2_OO· → ·CH_2_CH_2_OCH_2_OOH
R21	CH_3_CH_2_OCH(CH_3_)OO· → ·CH_2_CH_2_OCH(CH_3_)OOH
R22	CH_3_CH_2_OCH(C_2_H_5_)OO· → ·CH_2_CH_2_OCH(C_2_H_5_)OOH
1,6-H(s)	
R23	CH_3_(CH_2_)_2_OCH_2_OO· → CH_3_C·HCH_2_OCH_2_OOH
R24	CH_3_(CH_2_)_2_OCH(CH_3_)OO·· → CH_3_C·HCH_2_OCH(CH_3_)OOH
R25	CH_3_(CH_2_)_3_OCH_2_OO· → CH_3_CH_2_C·HCH_2_OCH_2_OOH
R26	CH_3_CH(CH_3_)CH_2_CH(CH_3_)OCH(CH_3_)OO· → CH_3_CH(CH_3_)C·HCH(CH_3_)OCH(CH_3_)OOH
1,6-H(t)	
R27	CH_3_CH(CH_3_)CH_2_OCH_2_OO· → CH_3_C·(CH_3_)CH_2_OCH_2_OOH
R28	CH_3_CH_2_CH(CH_3_)CH_2_OCH_2_OO· → CH_3_CH_2_C·(CH_3_)CH_2_OCH_2_OOH
R29	CH_3_CH_2_CH(CH_3_)CH(CH_3_)OCH_2_OO· → CH_3_CH_2_C·(CH_3_)CH(CH_3_)OCH_2_OOH
R30	CH_3_CH_2_CH(CH_3_)CH(CH_3_)OCH(CH_3_)OO· → CH_3_CH_2_C·(CH_3_)CH(CH_3_)OCH(CH_3_)OOH
1,7-H(p)	
R31	CH_3_CH_2_CH_2_OCH_2_OO· → ·CH_2_CH_2_CH_2_OCH_2_OOH
R32	CH_3_CH(CH_3_)CH_2_OCH_2_OO· → ·CH_2_CH(CH_3_)CH_2_OCH_2_OOH
R33	CH_3_CH(CH_3_)CH(CH_3_)OCH_2_OO· → ·CH_2_CH(CH_3_)CH(CH_3_)OCH_2_OOH
R34	CH_3_CH(CH_3_)CH(CH_3_)OCH(CH_3_)OO· → ·CH_2_CH(CH_3_)CH(CH_3_)OCH(CH_3_)OOH
1,7-H(s)	
R35	CH_3_(CH_2_)_3_OCH_2_OO· → CH_3_C·H(CH_2_)_2_OCH_2_OOH
R36	CH_3_CH_2_CH(CH_3_)CH_2_OCH_2_OO· → CH_3_C·HCH(CH_3_)CH_2_OCH_2_OOH
R37	CH_3_CH_2_CH(CH_3_)CH(CH_3_)OCH_2_OO· → CH_3_C·HCH(CH_3_)CH(CH_3_)OCH_2_OOH
R38	CH_3_CH_2_CH(CH_3_)CH(CH_3_)OCH(CH_3_)OO· → CH_3_C·HCH(CH_3_)CH(CH_3_)OCH(CH_3_)OOH
1,7-H(t)	
R39	CH_3_CH(CH_3_)CH_2_CH_2_OCH_2_OO· → CH_3_C·(CH_3_)(CH_2_)_2_OCH_2_OOH
R40	CH_3_CH(CH_3_)CH(CH_3_)CH_2_OCH_2_OO· → CH_3_C·(CH_3_)CH(CH_3_)CH_2_OCH_2_OOH
R41	CH_3_CH(CH_3_)CH(CH_3_)CH(CH_3_)OCH_2_OO· → CH_3_C·(CH_3_)CH(CH_3_)CH(CH_3_)OCH_2_OOH

**Table 2:** The maximum deviation of the energy barriers for intramolecular H–migration reaction of R^I^OR^II^OO· radicals (unit: kcal/mol).

Subclass	1,3-H	1,5-H	1,6-H	1,7-H
p	NA *^a^*	0.33	0.54	1.13
s	0.23	1.34	0.80	1.25
t	0.64	2.69	0.94	0.90
Avg *^b^*	1.16 *	7.19 *	6.85 *	6.70 *

The value with an asterisk in the last line is the maximum deviation of energy barriers for the reaction class. *^a^* NA represents there is no value for this reaction class. *^b^* Avg in the last line are the average energy barriers for reaction class.

**Table 3:** Comparison of the energy barriers by isodesmic reaction method with CBS–QB3 method (unit: kcal/mol).

Reaction Class	Reaction	CBS–QB3	IRM	Δ*V*
1,3-H(s)	R2	44.37	44.34	0.03
1,3-H(t)	R6	44.67	44.77	−0.10
1,5-H(p)	R11	23.08	23.02	0.06
1,5-H(s)	R14	21.98	22.21	−0.23
1,5-H(t)	R19	15.46	16.27	−0.81
1,6-H(p)	R21	27.39	27.40	−0.01
1,6-H(s)	R24	24.11	24.43	−0.32
1,6-H(t)	R28	20.04	20.74	−0.70
1,7-H(p)	R32	25.19	24.63	0.56
1,7-H(s)	R36	21.35	21.32	0.03
1,7-H(t)	R40	18.13	18.18	−0.05

Δ*V* represents the difference in energy barriers as determined by the isodesmic reaction method and the CBS–QB3 method.

**Table 4:** Comparison of the reaction enthalpies by isodesmic reaction method with CBS–QB3 method (unit: kcal/mol).

Reaction Class	Reaction	CBS–QB3	IRM	Curran et al. *^a^* [32]	Δ*H ^b^*
1,3-H(s)	R2	−46.33	−46.26	NA	−0.07
1,3-H(t)	R6	−46.33	−46.60	NA	0.27
1,5-H(p)	R10	NA	9.77	9.30	NA
1,5-H(p)	R11	12.16	12.34	NA	−0.18
1,5-H(t)	R19	10.53	10.21	NA	0.32
1,6-H(p)	R21	17.08	17.08	NA	0.00
1,6-H(s)	R24	14.29	14.08	NA	0.21
1,6-H(t)	R28	11.29	11.43	NA	−0.14
1,7-H(p)	R32	15.38	15.42	NA	−0.04
1,7-H(s)	R36	12.54	12.28	NA	0.26
1,7-H(t)	R40	9.76	9.46	NA	0.30

*^a^* The value of the reaction enthalpy from the work of Curran et al. [32] *^b^* Δ*H* represents the difference in reaction enthalpies as determined by the isodesmic reaction method and the CBS–QB3 method. NA represents that there is no value for this reaction class.

**Table 5:** Comparison of the rate constants at 500 K in this work with the analogous reactions in alkanes.

Reaction		R^I^OR^II^OO·	ROO·	
		This Work	Villano et al. *^a^* [7]	Bugler et al. *^b^* [34]
R10	COCOO· → ·COCOO	7.22	7.67 (1.06)	2.10 × 10^1^ (2.91)
R13	CCOCOO· → CC·OCOO	3.67 × 10^3^	2.84 × 10^2^ (12.92)	5.68 × 10^2^ (6.46)
R18	C_2_COCOO· → C_2_C·OCOO	8.61 × 10^5^	1.61 × 10^3^ (5.35 × 10^2^)	8.22 × 10^3^ (1.05 × 10^2^)
R20	CCOCOO· → ·CCOCOO	6.76 × 10^4^	3.21 (2.11 × 10^4^)	6.36 (1.06 × 10^4^)

*^a^* The ratios of average rate constants to the values of the corresponding average rate constants proposed by Villano et al. [7]. *^b^* The average rate constants to the values of the corresponding average rate constants proposed by Bugler et al. [34].

**Table 6:** Rate rules at high-pressure-limit for intramolecular H–migration reaction of R^I^OR^II^OO· radicals.

Reaction Class	Modified Arrhenius Parameters			500 K	1000 K
	*A* (s^−1^)	*n*	*E* (kcal/mol)	*f*	*f*
1,3-H(s)	1.36 × 10^9^	1.26	37.74	1.27	1.38
1,3-H(t)	9.58 × 10^9^	0.86	39.05	1.98	2.34
1,5-H(p)	2.03 × 10^13^	0.31	16.96	3.54 × 10^3^	2.43 × 10^3^
1,5-H(s)	4.47 × 10^9^	0.56	16.26	25.20	14.90
1,5-H(t)	1.22 × 10^10^	0.58	11.06	14.20	4.17
1,6-H(p)	1.19 × 10^13^	0.26	20.27	4.71 × 10^3^	2.63 × 10^3^
1,6-H(s)	2.15 × 10^9^	0.55	17.78	2.87	3.83
1,6-H(t)	2.62 × 10^9^	0.65	14.79	9.01	4.81
1,7-H(p)	7.37 × 10^12^	0.16	17.84	2.46 × 10^3^	1.59 × 10^3^
1,7-H(s)	2.32 × 10^9^	0.56	14.87	20.00	18.20
1,7-H(t)	2.19 × 10^10^	0.47	12.97	2.78	4.84

## Data Availability

Data are contained within the article and Supplementary Materials.

## Supplementary Materials

The following supporting information can be downloaded at https://www.mdpi.com/article/10.3390/molecules29184387/s1: The differences in energy barriers and reaction enthalpies between the M06–2X method and CBS–QB3 method for reference reactions are listed in Table S1. The energy barriers and reaction enthalpies for all reactions are listed in Table S2. The high-pressure-limit kinetic parameters (*A*, *n*, *E*) for all reactions are listed in Table S3. The full names of abbreviations related to this article are listed in Table S4.
