# Supplementary material for: Reaction Rate Rules of Intramolecular H-Migration Reaction Class for RIORIIOO·Radicals in Ether Combustion
Source: Molecules. 2024 Sep 15;29(18):4387. doi: 10.3390/molecules29184387 (PMC11433709; doi:10.3390/molecules29184387)
Supplement: Supplementary file 1 [file molecules-29-04387-s001.zip › molecules-3189834-supplementary.pdf]

Supporting Information

Reaction Rate Rules of Intramolecular H-migration Reaction Class for $R^{I}OR^{II}OO$•radicals in Ethers Ccombustion

Xiaohui Sun[1,*], Zerong Li[2]

[1]*School of Energy Engineering, Shanxi College of Technology, Shuozhou, 036000, China*

[2]*College of Chemistry, Sichuan University, Chengdu 610064, PR China*

*Correspondence: xiaohuisun526@foxmail.com

Content

(1) Energy barriers and reaction enthalpies

Table S1. Differences of energy barriers and reaction enthalpies between M06–2X method and CBS-QB3 method for reference reactions (unit: kcal/mol)

Table S2. Energy barriers and reaction enthalpies for all reactions (unit: kcal/mol)

(2) High-pressure-limit kinetic parameters

Table S3. High-pressure-limit kinetic parameters (*A, n, E*) for all reactions

(3) Noun interpretation

Table S4. The full names of abbreviations

(1) Energy barriers and reaction enthalpies

According to the reaction class-transition state theory, the first small reaction in each reaction subclass are chosen as reference reactions and other reactions in Table 1 are chosen as target reactions. The differences of the energy barriers and reaction enthalpies for reference reactions between the approximate method (M06–2X) and the accurate method (CBS–QB3) are calculated and the results are listed in Table S1. These differences from reference reactions are used as the corrections to energy barriers and reaction enthalpies for target reaction in the same reaction class.

Table S1. Energy barriers and reaction enthalpies for reference reactions (unit: kcal/mol)

Reaction class	Reaction	ΔV		$\Delta\Delta V^{\neq a}$	ΔH		$\Delta\Delta H^{b}$
		CBS-QB3	M06-2X		CBS-QB3	M06-2X	
1,3-H(s)	R1	44.48	48.37	-3.89	-46.20	-44.96	-1.24
1,3-H(t)	R5	45.34	48.72	-3.38	-47.30	-46.50	-0.80
1,5-H(p)	R10	23.35	26.01	-2.66	9.77	11.22	-1.45
1,5-H(s)	R13	22.40	24.88	-2.48	9.74	11.20	-1.46
1,5-H(t)	R18	18.96	22.05	-3.09	10.10	11.55	-1.45
1,6-H(p)	R20	26.92	28.22	-1.30	16.18	17.47	-1.29
1,6-H(s)	R23	24.92	23.63	-1.29	20.29	18.99	-1.30
1,6-H(t)	R27	21.90	20.61	-1.29	12.59	11.30	-1.29
1,7-H(p)	R31	25.51	24.22	-1.29	22.19	20.89	-1.30
1,7-H(s)	R35	22.55	21.26	-1.29	13.45	12.16	-1.29
1,7-H(t)	R39	19.23	17.93	-1.30	19.23	17.93	-1.30

[a]The difference of energy barriers between CBS−QB3 and M06−2X method. [b]The difference of reaction enthalpies between CBS−QB3 and M06−2X method.

Table S2. Energy barriers and reaction enthalpies for all reactions (unit: kcal/mol)

Reaction	ΔV^{a}	$\Delta V'^{b}$	ΔH^{c}	$\Delta H'^{d}$
1,3-H(s)				
R1	48.37	44.48	-44.96	-46.20

R2	48.23	44.34	-45.02	-46.26
R3	48.15	44.26	-39.27	-40.51
R4	48.13	44.25	-45.06	-46.30
1,3-H(t)				
R5	48.72	45.34	-46.50	-47.30
R6	48.15	44.77	-45.81	-46.60
R7	48.54	45.17	-46.40	-47.20
R8	48.74	45.36	-46.35	-47.15
R9	48.79	45.41	-46.44	-47.24
1,5-H(p)				
R10	26.01	23.35	11.22	9.77
R11	25.69	23.02	13.79	12.34
R12	25.81	23.14	13.93	12.48
1,5-H(s)				
R13	24.88	22.40	11.20	9.74
R14	24.68	22.21	17.22	15.76
R15	24.60	22.12	11.98	10.52
R16	24.95	22.48	12.14	10.68
R17	25.93	23.46	10.45	8.99
1,5-H(t)				
R18	22.05	18.96	11.55	10.10
R19	19.36	16.27	11.66	10.21
1,6-H(p)				
R20	28.22	26.92	17.47	16.18
R21	28.69	27.40	18.37	17.08
R22	28.75	27.46	18.45	17.15
1,6-H(s)				
R23	24.92	23.63	20.29	18.99
R24	25.73	24.43	15.38	14.08

R25	25.32	24.03	14.68	13.39
R26	25.56	24.26	12.61	11.32
1,6-H(t)				
R27	21.90	20.61	12.59	11.30
R28	22.04	20.74	12.72	11.43
R29	22.13	20.84	12.06	10.77
R30	22.84	21.55	12.93	11.64
1,7-H(p)				
R31	25.51	24.22	22.19	20.89
R32	25.93	24.63	16.71	15.42
R33	24.79	23.50	15.73	14.44
R34	25.35	24.06	16.66	15.36
1,7-H(s)				
R35	22.55	21.26	13.45	12.16
R36	22.61	21.32	13.57	12.28
R7	21.87	20.58	13.20	11.91
R38	23.12	21.83	13.18	11.89
1,7-H(t)				
R39	19.23	17.93	11.55	10.26
R40	19.47	18.18	10.75	9.46
R41	20.13	18.83	10.03	8.74

[a]The energy barriers before correction.

[b]The energy barriers after correction.

[c]The reaction enthalpies before correction.

[d]The reaction enthalpies after correction.

(2) High-pressure-limit kinetic parameters

Table S3. High-pressure-limit kinetic parameters (*A, n, E*) for all reactions

Reaction class	Reaction	Modified Arrhenius Parameters

		A(s^{-1})	n	E(kcal/mol)
**1,3-H(s)**				
	R1	2.14E+10	0.86	37.97
	R2	1.06E+09	1.33	37.83
	R3	6.52E+08	1.35	37.69
	R4	6.73e+08	1.36	37.64
**1,3-H(t)**				
	R5	1.86E+10	0.80	39.10
	R6	2.70E+09	0.92	38.59
	R7	1.16E+10	0.86	39.00
	R8	1.02E+10	0.85	39.20
	R9	1.03E+10	0.84	39.28
**1,5-H(p)**				
	R10	6.11E+13	0.31	16.95
	R11	1.30E+11	0.18	18.17
	R12	2.16E+09	0.67	17.00
**1,5-H(s)**				
	R13	1.30E+10	0.23	16.43
	R14	4.70E+09	0.35	16.35
	R15	1.91E+10	0.52	16.19
	R16	2.04E+09	0.67	16.56
	R17	2.82E+09	0.52	17.50
**1,5-H(t)**				
	R18	2.27E+10	0.51	13.30
	R19	1.68E+11	0.28	11.18
**1,6-H(p)**				
	R20	3.59E+13	0.26	20.27
	R21	4.77E+09	0.55	20.79
	R22	9.25E+08	0.69	20.85

**1,6-H(s)**				
	R23	6.01E+09	0.28	17.29
	R24	5.45E+09	0.55	18.09
	R25	4.13E+09	0.38	17.82
	R26	1.33E+09	0.58	17.86
**1,6-H(t)**				
	R27	7.78E+09	0.56	14.61
	R28	3.83E+09	0.65	14.94
	R29	1.22E+09	0.68	14.97
	R30	2.12E+09	0.61	15.80
**1,7-H(p)**				
	R31	2.96E+13	0.16	17.84
	R32	1.94E+09	0.52	18.23
	R33	2.01E+09	0.49	17.07
	R34	6.36E+09	0.42	17.78
**1,7-H(s)**				
	R35	4.15E+09	0.17	15.17
	R36	4.74E+09	0.51	15.30
	R37	5.91E+09	0.49	14.55
	R38	3.76E+09	0.51	15.67
**1,7-H(t)**				
	R39	6.89E+09	0.46	12.26
	R40	7.02E+09	0.51	12.38
	R41	1.13E+11	0.38	13.48

(3) Noun interpretation

Table S4. The full names of abbreviations

**CBS-QB3**	Complete Basis Set
**HEAT**	High accuracy Extrapolated Ab initio Thermochemistry

**CCSD(T)**	Coupled Cluster theory with Single and Double excitations and a quasi-perturbative treatment of con-nected Triple excitations
**cc-pvtz**	correlation-consistent polarized valence triple-ζ basis set
**B3LYP**	Becke's three-parameter hybrid method with Lee-Yang-Parr correlation correction
